# Lack of Cul4b, an E3 Ubiquitin Ligase Component, Leads to Embryonic Lethality and Abnormal Placental Development

**DOI:** 10.1371/journal.pone.0037070

**Published:** 2012-05-14

**Authors:** Baichun Jiang, Wei Zhao, Jupeng Yuan, Yanyan Qian, Wenjie Sun, Yongxin Zou, Chenhong Guo, Bingxi Chen, Changshun Shao, Yaoqin Gong

**Affiliations:** Key Laboratory of Experimental Teratology, Ministry of Education and Institute of Molecular Medicine and Genetics, Shandong University School of Medicine, Jinan, Shandong, China; Wellcome Trust Centre for Stem Cell Research, United Kingdom

## Abstract

Cullin-RING ligases (CRLs) complexes participate in the regulation of diverse cellular processes, including cell cycle progression, transcription, signal transduction and development. Serving as the scaffold protein, cullins are crucial for the assembly of ligase complexes, which recognize and target various substrates for proteosomal degradation. Mutations in human *CUL4B*, one of the eight members in cullin family, are one of the major causes of X-linked mental retardation. We here report the generation and characterization of *Cul4b* knockout mice, in which exons 3 to 5 were deleted. In contrast to the survival to adulthood of human hemizygous males with *CUL4B* null mutation, *Cul4b* null mouse embryos show severe developmental arrest and usually die before embryonic day 9.5 (E9.5). Accumulation of cyclin E, a CRL (CUL4B) substrate, was observed in *Cul4b* null embryos. *Cul4b* heterozygotes were recovered at a reduced ratio and exhibited a severe developmental delay. The placentas in *Cul4b* heterozygotes were disorganized and were impaired in vascularization, which may contribute to the developmental delay. As in human *CUL4B* heterozygotes, *Cul4b* null cells were selected against in *Cul4b* heterozygotes, leading to various degrees of skewed X-inactivation in different tissues. Together, our results showed that CUL4B is indispensable for embryonic development in the mouse.

## Introduction

Cullin-RING ligases (CRLs) complexes comprise the largest known class of ubiquitin ligases [1]. CRLs regulate diverse cellular processes, including cell cycle progression, transcription, signal transduction and development [2]. CRLs are multisubunit complexes composed of a cullin, RING protein and substrate-recognition subunit, which was linked by an adaptor. Human cullin family consists of eight members, CUL1, CUL2, CUL3, CUL4A, CUL4B, CUL5, CUL7 and PARC [3], among them, CUL4A and CUL4B have the highest degree of homology, with 83% identity in protein sequences [4]. There is only one ortholog, Cul4, in lower organisms. CUL4A CRL complexes contained Rbx1 and the adaptor protein DDB1. DDB1 interact with substrate recognition subunits, which determine the substrate specificity of the CUL4A CRL complexes [5], [6], [7], [8], [9]. The substrates of CUL4A CRL complexes include CDT1, p21, p27, p53, c-Jun, HOXA9, H3 and CHK1 that play important roles in cell cycle regulation, chromosome remodeling, and differentiation [10], [11].

Compared to CUL4A, CUL4B is less studied, and so far very few substrates of CUL4B CRL complexes have been identified [4], [12], [13], [14], [15]. However, mutations in human *CUL4B* appear to be a common cause of X-linked mental retardation (XLMR). To date, at least 12 families of XLMR have been reported to be attributable to base substitutions or deletions in *CUL4B* [16], [17], [18], [19], [20]. In addition to mental retardation, those patients also manifest short stature, abnormal gait, impaired speech and other abnormalities. These findings suggest that CUL4B and CUL4A do not necessarily play redundant roles during neurogenesis and other developmental processes.

**Figure 1:**
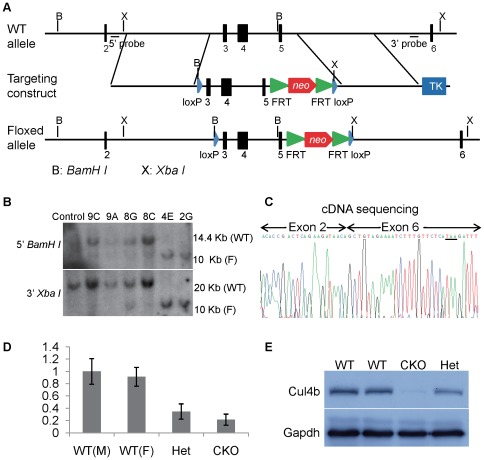
Generation of *Cul4b* flox mice. (A) Strategy for generation of *Cul4b* floxed targeting vector. On the top was shown the wild-type allele of *Cul4b* gene. The targeting vector and targeted allele were shown in the middle and at the bottom, respectively. *BamHI* (labeled B) and *XbaI* (labeled X) sites are indicated. Black bars indicating the positions of the probes used in Southern blots are also indicated. (B) Southern blot analysis of genomic DNA isolated from wild-type ES cells and selected ES cell clones. *BamHI* digested DNA was hybridized with 5′ probe and *XbaI* digested DNA was hybridized with 3′ probe. WT, wild-type allele; F, flox allele. (C) cDNA sequencing of *Cul4b* gene from the brain tissue of brain-specific knockout mice (*Cul4b* ^flox/Y^;*Nesin-Cre*). As predicted, exon 2 was spliced onto exon 6 after the excision of exons 3–5. (D) Real-time RT-PCR analysis of *Cul4b* mRNA isolated from brain tissues of wild-type males (*Cul4b* ^+/Y^;*Nestin-Cre* ^+/−^), wild-type females (*Cul4b^+/+^*;*Nestin-Cre* ^+/−^), heterozygous females (*Cul4b* ^+/flox^;*Nestin-Cre* ^+/−^), and conditional knock-out males (*Cul4b* ^flox/Y^;*Nestin-Cre* ^+/−^). (E) Western blot analysis of Cul4b protein isolated from brain tissues of wild-type, heterozygous and conditional knockout mice using an anti-Cul4b antibody. Gapdh was used as a loading control.

While *Cul4a* knockout mice have been independently generated by several groups, they showed highly variable phenotypes depending on the nature of the mutation introduced. Homozygous deletion of exon 1 of *Cul4a* was first reported to result in embryonic lethality [21]. However, a recent study showed that deletion of exon 1 of *Cul4a* inadvertently deleted the essential *Pcid2* gene located adjacent to *Cul4a* on the complementary strand [22]. Mice with deletion of exons 17–19 of *Cul4a*, on the other hand, were viable and displayed no overt developmental abnormalities, although skin-specific *Cul4a* ablation rendered resistance to UV-induced skin carcinogenesis [22]. Similarly, homozygous deletion of exons 4–8 of *Cul4a* resulted in no gross abnormalities [23]. However, the male knockout mice were sterile and exhibit severe deficiencies in spermatogenesis [23], [24]. Homozygous deletion of exons 4–8 of *Cul4a* is also associated with severe proliferation defects in embryonic fibroblasts and hepatocytes, and an increase in genome instability [25].

In this study, we generated *Cul4b* floxed mice and crossed it to *EIIa-Cre* transgenic mice to produce *Cul4b* null mice. We observed that *Cul4b* null mice are embryonic lethal. *Cul4b* null embryos displayed decreased proliferation and increased apoptosis. *Cul4b* heterozygotes were also affected, as reflected by their recovery at a reduced ratio at birth and by their developmental delay. Cells expressing *Cul4b* null allele in *Cul4b* heterozygous mice were selected against, to different degrees in different tissues, from early embryogenesis to early postnatal development. The embryonic lethality of *Cul4b* null mice, when compared to the lack of gross abnormalities in *Cul4a* null mice, indicated that *Cul4b* has diverged from *Cul4a* to carry out some essential and unique functions during embryogenesis.

## Results

### Generation of *Cul4b* floxed mice

Because CUL4B-deficient cells are strongly selected against [17], we envisaged that it might be difficult to generate Cul4b-deficient embryonic stem (ES) cells via conventional knockout technology. We therefore used the Cre/loxP strategy to generate *Cul4b* floxed ES cells. First, a *Cul4b* floxed targeting vector was constructed (Fig. 1A). In this vector, exons 3–5 were floxed by two loxP sites. A *neo* gene flanked by two FRT sites and a *TK* gene were also insert into intron 5 and vector backbone, respectively, for ES cells selection.

The targeting vector was linearized and electroporated into 129 male ES cells (RW.4) for homologous recombination. Ninety six clones were screened by long-range PCR (data not shown) and Southern blot (Fig. 1B). Two correctly targeted clones, 2G and 4E, were identified. Targeted ES cells were injected into C57BL/6J blastocysts to produce chimaeric mice, which were then used for germline transmission to produce *Cul4b* floxed mice.

Neither male hemizygous (*Cul4b* ^flox^/Y) nor female heterozygous/homozygous (*Cul4b* ^+/flox^/*Cul4b* ^flox/flox^) for the *Cul4b* floxed allele showed any apparent phenotype, suggesting that the flox allele did not disturb the normal function of *Cul4b* gene. To verify that *Cul4b^flox^* can be rendered nonfunctional by the expression of Cre, due to the removal of exons 3–5, we generated brain-specific knockout mice *Cul4b* ^flox/Y^;*Nestin-Cre* mice and sequenced the cDNA of *Cul4b* prepared from the brain. We observed that exon 2 was indeed spliced onto exon 6, as a result of removal of exons 3–5 (Fig. 1C). The deletion would also result in a frameshift, generating 8 missense condons followed by a stop codon (Fig. 1C, underlined). The mutant allele is thus predicted to generate a peptide of merely 28 amino acids, if it can be expressed. Thus, deletion of exons 3–5 created a null mutation. Furthermore, the *Cul4b* mRNA level in brains of the conditional knockout mice is much lower than that of littermate wild-type control (Fig. 1D), suggesting that the truncation mutation also resulted in nonsense-mediated decay (NMD), as observed in patients with *CUL4B* nonsense mutation [17]. Western blot analysis showed that Cul4b protein was nearly absent in brain tissues of conditional knockout mice (Fig. 1E). Together, these results indicate that deletion of exons 3–5 in *Cul4b* results in a null mutation.

### *Cul4b* null mice are not viable

To investigate the function of *Cul4b* during mouse development, *Cul4b* floxed mice were crossed to *EIIa-Cre* transgenic mice to generate constitutive *Cul4b* null mice. The Cre transgene in the *EIIa-Cre* mice is driven by the adenovirus *EIIa* promoter and is expressed in a wide range of tissues, including germ cells. Because *EIIa-Cre* is not expressed in all cells (mosaic expression), *Cul4b* ^+/flox^ females were first crossed to *EIIa-Cre* ^+/+^ males to obtain mosaic females that are *Cul4b* ^+/flox^/*Cul4b* ^+/null^;*EIIa-Cre* ^+/−^. Mosaic females were then crossed to wild-type males to generate progeny of *Cul4b* knockout male mice (*Cul4b* ^null^/Y), *Cul4b* heterozygous females (*Cul4b* ^+/null^) and wild-type mice (*Cul4b* ^+/+^ and *Cul4b* ^+^/Y). While *Cul4b* ^+/null^ females were recovered, no *Cul4b* ^null^/Y mice were present in the weaned pups (Table 1), suggesting that *Cul4b* ^null^/Y conceptuses can not develop to term or die before weaning.

**Table 1:** Distribution of *Cul4b* genotypes in progeny of *Cul4b* ^+/flox^/*Cul4b* ^+/null^;*EIIa-Cre* ^+/−^ females.

Group	Wild-Type	Heterozygous	Knockout	Absorbed
*Cul4b* genotype	*Cul4b* ^+/+^ or *Cul4b* ^+/Y^	*Cul4b* ^+/null^	*Cul4b* ^null/Y^	ND^a^
Expected Mendelian %	50%	25%	25%	-
3 weeks (%) (n = 135)	107 (79%)	28 (21%)	0 (0%)	-
14.5 dpc (%) (n = 61)	31 (51%)	11 (18%)	0 (0%)	19 (31%)
12.5 dpc (%) (n = 39)	17 (44%)	9 (23%)	0 (0%)	13 (33%)
10.5 dpc (%) (n = 38)	19 (50%)	12 (32%)	0 (0%)	7 (18%)
9.5 dpc (%) (n = 31)	15 (48%)	7 (23%)	0 (0%)	9 (29%)
8.5 dpc (%) (n = 28)	15 (54%)	6 (21%)	7 (25%)	(0%)

Litters were dissected at the times shown and genotyped by PCR as described in Materials and Methods.

a ND indicates that the *Cul4b* genotype could not be determined by PCR.

### *Cul4b* null embryos showed early developmental arrest

**Figure 2:**
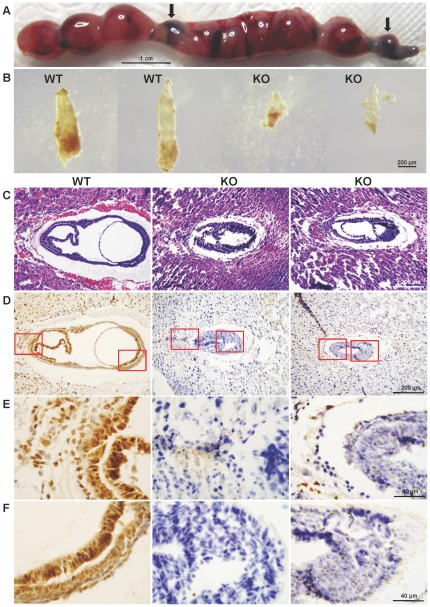
Morphology and histology of *Cul4b* null embryos. (A) Uterus excised from pregnant female at 12.5 dpc. Arrows indicate absorbed embryos. (B) Photomicrographs of E7.5 embryos dissected from surrounding deciduas tissue of the same uterus. The two embryos on the left appear normal in size and morphology and the two on the right were much smaller and were partially deteriorated. The bar represents 200 µm. (C) H&E staining of paraffin sections of wild-type and *Cul4b* null embryos at 7.5 dpc. Littermate embryos at 7.5 dpc were paraffin embedded and cross sectioned together with their surrounding deciduas. The genotype of each embryo was determined by immunohistochemistry using an anti-Cul4b antibody, as shown below. (D) Immunohistochemistry of paraffin sections of wild-type and *Cul4b* null embryos at 7.5 dpc with an anti-Cul4b antibody. (E–F) Photomicrographs with higher magnification of the stained section shown in (D).

To determine the exact time point when *Cul4b* ^null^/Y conceptuses die, timed mating was performed and embryos at different developmental stages were dissected. DNA was extracted from yolk sac or total embryo and used for PCR genotyping. Genotyping of E9.5, 10.5, 12.5 and 14.5 embryos showed that no *Cul4b* ^null^/Y conceptuses were recovered on 9.5 day post coitum (dpc) and beyond. Instead, a proportion of embryos, ∼25%, appeared to have been absorbed and only placentas or empty deciduas remained (Fig. 2A), suggesting that these embryos implanted but died by E9.5. *Cul4b* null embryos were recovered at a ratio expected of Mendelian inheritance at E7.5 and 8.5, and no empty deciduas were found at these developmental stages. However, the *Cul4b* null embryos were much smaller (Fig. 2B). The recovery of *Cul4b* null embryos at the expected Mendelian ratio indicated that the mosaic females are functionally *Cul4b* ^+/null^ in their capacity to transmit *Cul4b* ^null^ allele.

To gain further insight into the anomalies in the *Cul4b* null embryos, we performed H&E staining of paraffin-embedded sections of E7.5 embryos. While in wild-type embryos, ectoderm, mesoderm and endoderm appeared to have properly formed, the embryonic part of the cylinder was not expanded and boundaries between germ layers were indiscernible in the *Cul4b* null embryos (Fig. 2C), which were identified by their negative staining by anti-Cul4b antibody (see below). In addition, extraembryonic compartment in *Cul4b* null embryos was also underdeveloped and poorly organized (Fig. 2C). Thus, in general, the *Cul4b* null embryos are developmentally retarded when compared to stage-matched wild-type embryos.

Immunohistochemistry, using an anti-Cul4b antibody, showed that Cul4b was ubiquitously expressed in wild-type E7.5 embryos (Fig. 2D–2F), including ectoplacental cone, trophoblast giant cells, chorion (Fig. 2E), and the embryonic region (Fig. 2F). Expression of *Cul4b* in extraembryonic regions suggests that Cul4b may be involved in placental development. As expected, the *Cul4b* null embryos were devoid of staining by anti-Cul4b.

Because a high degree of homology exists between *Cul4a* and *Cul4b*, we also examined the expression of *Cul4a* in 7.5 dpc embryos. Positive staining of Cul4a was detected throughout the whole embryos (Figure S1A). Furthermore, the expression of Cul4a was not found to differ between *Cul4b* null and wild-type embryos, suggesting that deletion of *Cul4b* gene did not affect the expression of *Cul4a* gene. Apparently, *Cul4a* failed to compensate for the lack of *Cul4b* in early embryonic development.

### Decreased proliferation and increased apoptosis in *Cul4b* null embryos

**Figure 3:**
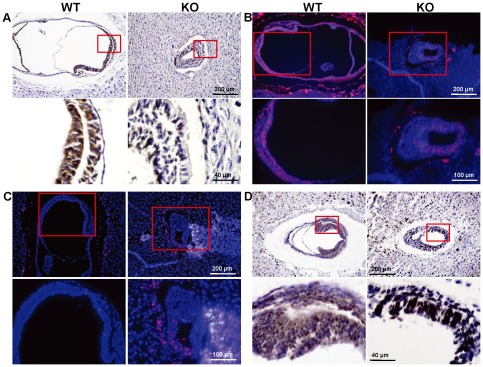
Decreased proliferation and increased apoptosis in *Cul4b* null embryos. (A) Paraffin sections of wild-type and *Cul4b* null embryos at 7.5 dpc were stained with an antibody against Ki67, a proliferation marker. Sections were counterstained with haematoxylin. Lower panels are the higher magnification of the upper panels. (B) Paraffin sections of wild-type and *Cul4b* null embryos at 7.5 dpc were analyzed by immunostaining against BrdU, and counterstained with DAPI. Lower panels are the higher magnification of the upper panels. (C) Paraffin sections of wild-type and *Cul4b* null embryos at 7.5 dpc were analysed by TUNEL assay for labeling apoptotic cells, and counterstained with DAPI. Lower panels are the higher magnification of the upper panels. (D) Paraffin sections of wild-type and *Cul4b* null embryos at 7.5 dpc were stained with an antibody against cyclin E. Sections were counterstained with haematoxylin. Lower panels are the higher magnification of the upper panels.

To investigate what causes the developmental delay of the *Cul4b* null embryos, we analyzed the proliferative activity and apoptosis in E7.5 embryos. The proliferation was evaluated by immunohistochemical staining of paraffin sections of E7.5 embryos for Ki67, a marker of proliferating cells. While there was a high level of proliferation in the wild-type embryos, proliferative cells were less abundant in *Cul4b* null embryos (Fig. 3A). BrdU incorporation assay confirmed the decrease in proliferation in *Cul4b* null embryos (Fig. 3B), suggesting that the proliferative activity was greatly compromised in *Cul4b* null embryos.

Apoptosis in E7.5 embryos was evaluated by TdT-mediated dUTP nick end labeling (TUNEL) assay. Apoptotic cells were rarely detected in wild-type E7.5 embryos; however, the number of apoptotic cells was remarkably increased in the *Cul4b* null embryos (Fig. 3C). Apoptotic cells were also observed to line along the Reichert's membrane.

It was previously reported that CUL4B can interact with cyclin E and the CUL4B immunocomplexes can polyubiquitinate cyclin E *in vitro* [26]. We previously showed that silencing of *CUL4B* could lead to increased accumulation of cyclin E [4]. To determine whether deficiency of Cul4b would also cause increased accumulation of cyclin E in the *Cul4b* null embryos, the cyclin E level was examined using immunohistochemistry. Indeed, cyclin E was accumulated in *Cul4b* null embryos compared to littermate wild-type embryos (Fig. 3D).

The levels of other known substrates of CRL4 complex, p27 and p53, were also examined in wild-type and *Cul4b* null embryos at 7.5 dpc. They were not found to differ between *Cul4b* null embryos and wild-type embryos (Figure S1B–S1C), suggesting that Cul4b probably does not play a critical role in the proteosomal degradation of those proteins during embryonic development. Alternatively, Cul4a may have compensated for the lack Cul4b in lowering the levels of those substrates.

Taken together, these results suggested that the developmental delay in *Cul4b* null embryos is attributable to a reduction in proliferation and an increase in apoptosis.

### Reduced recovery and growth retardation of *Cul4b* heterozygous mice

**Figure 4:**
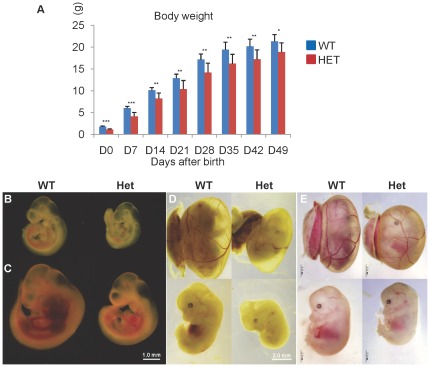
Growth retardation of *Cul4b* heterozygous mice during embryonic development. (A) Bodyweights of *Cul4b* heterozygous mice and littermate wild-type females after birth. Data were presented as mean±SD. N = 8, *: p<0.05; **: p<0.01; ***: p<0.001. (B–E) Representative photographs of *Cul4b* heterozygous embryos and littermate wild-type controls at 9.5 (B), 10.5 (C), 12.5 (D) and 14.5 (E) dpc. The bar represents 1 mm in (B–C) and 2 mm in (D) and (E), respectively.

While no *Cul4b* null conceptuses could survive to E9.5, the *Cul4b* heterozygous females were also recovered at a lower than expected ratio at weaning (Table 1). This deficit was probably caused by an increased prenatal lethality in *Cul4b* heterozygotes, because their ratio was reduced even at E14.5. For the *Cul4b* heterozygotes that survive to term, they were significantly smaller than their wild-type littermates. The average body weight of heterozygous newborns (1.13±0.17 g) was much smaller than that of wild-type controls (1.79±0.12 g). However, *Cul4b* heterozygous mice were able to gradually catch up after birth, and the body weight difference became narrow in adults (Fig. 4A). Except for growth retardation, *Cul4b* heterozygous mice showed no gross abnormalities for the first 18 months.

The fact that *Cul4b* heterozygous mice were smaller in size at birth but could catch up gradually suggests that developmental delay is primarily due to factor(s) that operate during prenatal development. Therefore, heterozygous embryos at different development stages were dissected and examined (Fig. 4B–4E). *Cul4b* heterozygous embryos were smaller than wild-type controls at all development stages examined, suggesting that growth retardation began at early developmental stage. In addition, the *Cul4b* heterozygous embryos appeared pale (Fig. 4D and 4E). Blood vessels on yolk sac were not well developed (Fig. 4D), and the blood flow appeared to be reduced (Fig. 4E). Poor vascularization and shortage in blood supply may have caused undernourishment in the heterozygous embryos.

### Placental defects of *Cul4b* heterozygous embryos

**Figure 5:**
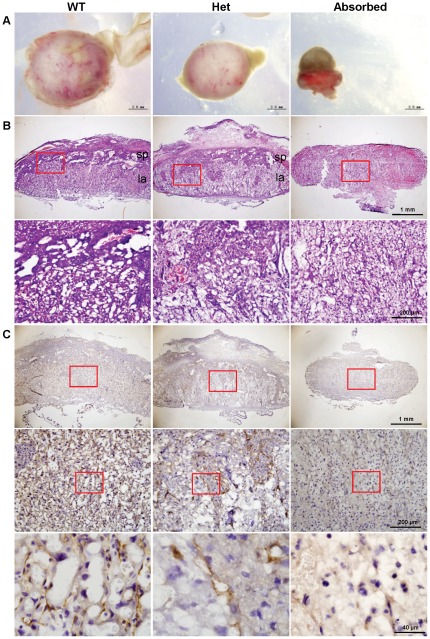
Morphology and histology of placentas of wild-type, *Cul4b* heterozygous and absorbed embryos at 14.5 dpc. (A) Representative photographs of placentas of wild-type, *Cul4b* heterozygous and absorbed embryos at 14.5 dpc. (B) H&E staining of radial sections of placentas. sp, spongiotrophoblast layer; la, labyrinthine layer. Lower panels are the higher magnification of the upper panels. (C) Immunohistochemisty of radial sections of placentas with an antibody to PECAM, an angiogenesis marker. Middle panels are the higher magnification of the upper panels, and lower panels are the higher magnification of the middle panels.

To further delineate the mechanism underlying the growth retardation of *Cul4b* heterozygotes during prenatal development, we examined the morphology and histology of placentas at E14.5. Placentas of *Cul4b* heterozygous embryos appeared smaller and paler than those of wild-type (Fig. 5A). H&E staining of placental sections showed that the labyrinth layer of *Cul4b* heterozygous placentas was more loosely formed compared to that of wild-type controls, and was deeply invaded by the spongiotrophoblast layer, leaving the demarcation between labyrinth and spongiotrophoblast layers indiscernible (Fig. 5B, upper panels). While the network in the labyrinth layer of placentas in the wild-type was highly compacted and densely branched; the labyrinth layer in *Cul4b* heterozygotes was laid out very loosely (Fig. 5B, lower panels). The labyrinth and spongiotrophoblast layers were indistinguishable in the placentas of absorbed embryos.

The vascular network in placenta was further characterized by immunohistochemistry using an antibody to platelet/endothelial cell adhesion molecule-1 (PECAM), which marks endothelial cells in blood vessels. In contrast to normal placentas in which the blood vessels were well formed and uniformly distributed (Fig. 5C, left panels), PECAM staining signals in *Cul4b* heterozygous placentas only appeared in rather isolated regions (Fig. 5C, middle panels). Furthermore, vascular structures were not readily recognizable within the PECAM-positive islands. Only background staining of PECAM was detected in placentas of absorbed embryos (Fig. 5C, right panels).

Taken together, these results showed that the placentas of *Cul4b* heterozygous embryos were generally disorganized and were impaired in vascularization, which may contribute to the growth retardation during prenatal development of *Cul4b* heterozygous mice.

### Pattern of X-chromosome inactivation (XCI) in *Cul4b* heterozygous mice

**Figure 6:**
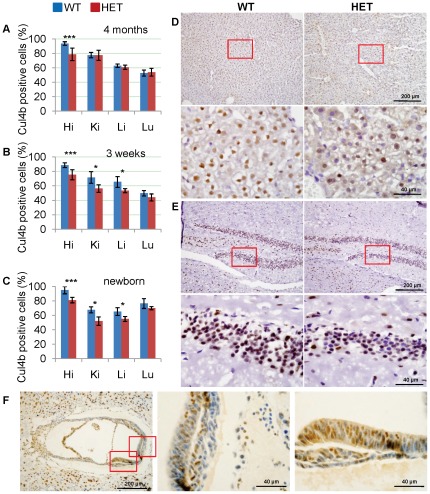
Characterization of X chromosome inactivation by Cul4b expression in heterozygous mice. (A–C) Percentages of cells positive for Cul4b of *Cul4b* heterozygous mice and littermate wild-type female controls at 4 months (A), 3 weeks (B) and newborn (C). More than 2,000 cells of each tissue were scored. Hi, hippocampus; Ki, kidney; Li, liver; Lu, lung. Data were presented as mean±SD. *: p<0.05; **: p<0.01; ***: p<0.001. (D–E) Representative images of liver (D) and hippocampus (E) at 3 weeks stained with an antibody against Cul4b. Sections were counterstained with haematoxylin. Lower panels are the higher magnification of the upper panels. (F) Immunohistochemistry of paraffin sections of *Cul4b* heterozygous embryos at 7.5 dpc with an anti-Cul4b antibody. Embryos at 7.5 dpc were paraffin embedded and cross sectioned together with their surrounding deciduas. Middle and right panels are the higher magnification of the left panel.

Because *Cul4b* is X-linked, *Cul4b* heterozygous females are functional mosaics in terms of the expression of *Cul4b*. The somatic cells are either *Cul4b* functional or null depending on the choice of the X-chromosome that becomes inactivated. In the XLMR family with *CUL4B* mutation, X-chromosome inactivation (XCI) was extremely skewed in peripheral blood cells of carriers, due to the selection that favors the cells in which the mutant *CUL4B* allele is inactivated [17], [20]. We first determined the XCI pattern in 4-month-old adult *Cul4b* heterozygous females by immunohistochemical analysis for *Cul4b* expression. If XCI were balanced in *Cul4b* heterozygous females, the proportion of cells expressing *Cul4b* should be ∼50% of that in wild-type females. On the other hand, if XCI is skewed toward *Cul4b* allele, as in human *CUL4B* heterozygous carriers, the percentage of cells expressing *Cul4b* should be closer to that in wild-type females. As shown in Fig. 6A, the percentages of cells expressing *Cul4b* in kidney, liver and lung were identical between *Cul4b* heterozygous females and wild-type females, suggesting that XCI is extremely skewed in those organs. XCI in hippocampus was also skewed, but to a lesser extent, since the percentage of *Cul4b* positive cells in heterozygotes remained lower than that in wild-type. Western blot assay showed a similar trend (Figure S2). While the expression levels of Cul4b in liver and lung in *Cul4b* heterozygous mice were comparable to those in wild type mice, the expression levels in cortex and hippocampus were decreased in *Cul4b* heterozygous mice compared to those in wild type mice.

The skewed XCI toward *Cul4b* ^null^-bearing chromosome could be caused either by an initiation of XCI that favors the *Cul4b* ^null^-bearing X or by a selection against *Cul4b* ^null^-expressing cells during development. To distinguish between those two possibilities, we next examined the XCI pattern in younger *Cul4b* heterozygous females. As shown in Fig. 6B–C, the percentages of *Cul4b*-expressing cells in lung were not significantly different between *Cul4b* heterozygous and wild-type females even in the newborn. However, the percentages of Cul4b positive cells in kidney and liver were significantly lower in heterozygotes than in wild-type (Fig. 6B–C). These results suggest that the skewed XCI is caused by a gradual selection against the Cul4b negative cells and that different organs face differential selection pressure during prenatal and postnatal development. While *Cul4b* null cells in lung were nearly all eliminated before birth, those cells remained in hippocampus four months after birth (Fig. 6A–C, 6E). The less stringent selection against *Cul4b* null cells in hippocampus is consistent with the clear difference in the *Cul4b* expression level between *Cul4b* heterozygotes and wild-type in the brain (Fig. 1D and 1E, Fig. S2).

To gain insight into the XCI pattern at embryonic stage, we examined the distribution of Cul4b-positive and –negative cells in E7.5 embryos. XCI is usually completed by E6.5 in epiblasts. As shown in Fig. 6F, Cul4b-positive and –negative cells were not evenly distributed in the embryonic regions of the E7.5 heterozygous embryos. While the two types of cells appeared in an intermingled pattern and in roughly equal proportions in some areas (middle), indicative of the randomness of XCI before selection took place, Cul4b-positive cells were predominant in other areas (right), suggesting that selection for Cul4b-positive cells started before E7.5 wherein. The differential selection in different embryonic regions at early developmental stage may contribute to the distribution pattern of Cul4b-negative cells and the selection dynamics in various tissues at later developmental stages.

## Discussion

The cullin members are critical for the assembly of CRL complexes that play important roles in multiple cellular processes. Lack of cullin function has indeed been shown to have severe consequences in cellular function and organism development. Disrupting either *Cul1* or *Cul3* genes in mice caused embryonic lethality and dysregulation of cyclin E [27], [28], [29]. Deletion of *Cul7* caused neonatal lethality, growth retardation and vascular abnormality [30]. In this study, we showed that deletion of *Cul4b* resulted in embryonic lethality in null hemizygotes. This finding is consistent with the embryonic lethality of *Cul4b* mutant mice that were generated by gene trap technology [31]. In addition, *Cul4b* heterozygotes were recovered in deficit and those that survive to term exhibited a developmental delay. Underscoring the importance of Cul4b function is the observed skewedness of X-inactivation in *Cul4b* heterozygotes as a result of selection against *Cul4b* null cells. The severe phenotype of *Cul4b* null mice is in a sharp contrast to the lack of obvious phenotype in *Cul4a* null mice, although the two cullin members are closely related. These findings with *Cul4b* mutant mice, together with the development of XLMR syndrome in humans carrying *CUL4B* mutation, suggest that CUL4A cannot fully compensate for the absence of CUL4B activity in mammals.

While mutations in *CUL4B* cause mental retardation, short stature, impaired speech, and other abnormalities, the patients usually survive to adulthood. Thus, the *Cul4b* mutant mice are more severely affected. The mechanism underlying this species-specific difference in phenotypic severity caused by CUL4B deficiency remains to be elucidated. It is possible that certain mutations in humans may retain residual function of CUL4B and thus have less deleterious effect than the *Cul4b* null mutation in the mouse. However, several mutations in human *CUL4B* resemble null mutations. The p.R388X mutation, for example, would have truncated the whole CULLIN domain and the rest of the C-terminus [16], [17]. Moreover, this mutation caused nonsense mediated decay of *CUL4B* mRNA [17]. Two reported deletions in *CUL4B* causing XLMR would have been devoid of most of the CULLIN domain, which is essential for CRL activity [19], [20]. It should be noted that the more severe phenotype in mouse mutants of genes responsible for XLMR is not restricted to *Cul4b* null mice. Loss of *Atrx* function, which is the second most common cause of XLMR, next to fragile X gene, also resulted in early embryonic lethality in mice [32], and even deletion of *Atrx* just in forebrain caused perinatal lethality [33]. Targeted inactivation of *Huwe1* in the CNS also resulted in neonatal lethality [34]. It is also possible that certain functions of CUL4B are redundantly carried out by CUL4A in the humans, but not in the mouse. We are tempted to speculate that because extraembryonic development is particularly affected in *Cul4b* null and heterozygous embryos, CUL4B is probably more critical for placental development in the mouse than in the humans. Future studies employing ablation of *Cul4b* in embryo proper may help resolve this issue.

We previously showed that silencing of *CUL4B* led to an increased accumulation of cyclin E and a reduced cell proliferation that was accompanied by a prolonged S phase [4]. Correspondingly, we found that *Cul4b* null embryonic cells are also accompanied by an increased accumulation of cyclin E. The inverse relationship between CUL4B and cyclin E was also observed in mouse liver in which *Cul4b* was ablated (unpublished data). While several recent studies showed that excessive cyclin E may play negative roles in cell proliferation [35], [36], it remains to be determined whether the developmental arrest in *Cul4b* null embryos is caused by excessive cyclin E.

Unexpectedly, we observed that *Cul4b* heterozygous mice were also affected, as reflected by their recovery at a lower than expected ratio and by their remarkable developmental delay at birth. They began to catch up with their wild-type littermates after birth. This delay was probably caused by the disorganization and impaired vascularization in placenta. These data suggested that *Cul4b* played an important role in placental development, which is crucial for nutrition and oxygen supply to the developing embryos. Indeed, *CUL7*, another cullin family member, was found to be over-expressed in placentas associated with intra-uterine growth restriction [37]. Furthermore, targeted disruption of *Cul7* gene resulted in abnormal vascular morphogenesis in placenta [30], a phenotype similar to what we observed in *Cul4b* heterozygous embryos. This developmental delay in *Cul4b* heterozygous mice is in contrast to human carriers, whose body sizes are in normal range in their childhood [17]. While it is possible that the proliferative disadvantage of the *Cul4b* null cells during late prenatal development may also contribute to the developmental delay of *Cul4b* heterozygous females, we think it less likely because selection against *Cul4b* null cells started rather early during embryogenesis, and the *Cul4b* functional cells would quickly compensate for the loss of the *Cul4b* null cells.

As reported for *Atrx* heterozygous mice in which selection against *Atrx* null cells followed different dynamics in different tissues [38], the selection pressure against *Cul4b* null cells also varies among different organs. While the percentages of *Cul4b*-expressing cells in lung were not significantly different between *Cul4b* heterozygous and wild-type females even in the newborn, a significant proportion of *Cul4b* null cells still lingered in hippocampus four months after birth. The different selection pressure of *Cul4b* null cells may reflect differential requirement of Cul4b function in proliferation and maintenance in various organs.

In summary, we showed that *Cul4b* null mice were embryonic lethal at around 8.5 dpc. *Cul4b* null embryos showed a reduction in proliferative capacity and an increase in apoptosis. *Cul4b* heterozygous mice exhibited prenatal and early postnatal growth retardation that may be related to defective placental development. Our results illustrate the functional importance of *Cul4b* gene in mouse embryonic development. The *Cul4b* floxed mice should serve as a powerful tool for future study of *Cul4b* function in various cellular processes and in various tissues.

## Materials and Methods

### Generation of mice with the floxed *Cul4b* gene

The animal work was approved by Animal Use Committee, Shandong University School of Medicine (Approval number: ECAESDUSM2008005). *Cul4b* floxed mice were generated at National Resource Center of Mutant Mice/Model Animal Research Center of Nanjing University. First, a *Cul4b* floxed targeting vector was constructed. Briefly, a 14.5 Kb DNA fragment of the mouse *Cul4b* gene, spanning the region from intron 2 to intron 5, was subcloned from a BAC clone (BMQ-455M17) into a vector which contained a TK cassette. A loxP site was inserted into intron 2 of *Cul4b* gene and a FRT-neo-FRT-loxP cassette was inserted into intron 5. (Fig. 1A)

The floxed targeting vector was linearized using *NotI* restriction enzyme and electroporated into 129 male ES cells (RW.4, obtained from ATCC), followed by selection in culture medium containing G418 and ganciclovir. To identify correctly targeted clones, 96 clones were selected, replicated and screened by long-range PCR using DNA isolated from each clone. Positive clones identified were further confirmed by Southern blot analysis. Southern blot of DNA digested with *BamHI* restriction enzyme with the 5′ probe that hybridizes with the upstream of the targeted region yielded a 14.4 kb fragment for the wild-type allele and a 10 kb fragment for the targeted allele. Analysis using *XbaI* restriction enzyme with the 3′ probe that hybridizes with the downstream of the target region revealed a 20 kb fragment for the wild-type allele and a 10 kb fragment for the targeted allele.

Correctly targeted ES cells were injected into the blastocysts of E3.5 embryos from hyperovulated C57BL/6J mice. Surviving blastocysts were transferred into the oviducts of pseudopregnant recipient females to produce chimaeric mice, which were further crossed with wild-type mice for germline transmission to produce *Cul4b* floxed mice.

### Generation of constitutive and brain-specific *Cul4b* knock-out mice

To produce mice null for *Cul4b* gene, *Cul4b* floxed mice were crossed with *EIIa-Cre* transgenic mice [39], in which Cre transgene was under the control of the adenovirus *EIIa* promoter that drives the expression of Cre recombinase in a wide range of tissues, including the germ cells that transmit the genetic alteration to progeny. Due to the mosaic pattern of Cre expression in *EIIa-Cre* transgenic mice, deletion of the floxed fragment is usually not achieved in all the somatic cells, thus commonly resulting in genetically mosaic mice. To avoid mosaicism, the following strategy was used. *Cul4b* ^+/flox^ females were crossed with *EIIa-Cre* ^+/+^ males to obtain mosaic females that are *Cul4b* ^+/flox^/*Cul4b* ^+/null^;*EIIa-Cre* ^+/−^. Female mosaic mice were subsequently crossed with wild-type male mice. A germ cell produced by the mosaic may carry either *Cul4b* ^null^ or *Cul4b* ^flox^ allele, but not both. The *Cul4b* ^null^ type of germ cells would give rise to progeny that were *Cul4b* knockout males (*Cul4b* ^null/^Y) and *Cul4b* heterozygous females (*Cul4b* ^+/null^). The *Cul4b* ^flox^ type of germ cells, on the other hand, will give rise to *Cul4b* ^flox/^Y males or *Cul4b* ^+/flox^ females. As shown in the Results, even though the females were supposed to be *Cul4b* ^+/flox^/*Cul4b* ^+/null^;*EIIa-Cre* ^+/−^, no *Cul4b* ^flox^ allele was detected in the progeny, suggesting that *Cul4b* ^flox^ allele is also converted into *Cul4b* ^null^ during germline transmission.

To produce conditional knock-out mice in which *Cul4b* gene was specific deleted in brain tissue, *Cul4b* ^+/flox^ female mice were cross with *Nestin-Cre* transgenic mice [40], in which Cre recombinase was under the control of the promoter and enhancer of rat *nestin* that was primarily expressed in the nervous system. These crosses would be expected to yield wild-type females (*Cul4b^+/+^*;*Nestin-Cre* ^+/−^), heterozygous females (*Cul4b* ^+/flox^;*Nestin-Cre* ^+/−^), wild-type males (*Cul4b* ^+/Y^;*Nestin-Cre* ^+/−^), and conditional knock-out males (*Cul4b* ^flox/Y^;*Nestin-Cre* ^+/−^) in a ratio of 1∶1∶1∶1.

The *EIIa-Cre* and *Nestin-Cre* transgenic mice were purchased form model animal research center of Nanjing University. All experiments involving animals were conducted in compliance with national regulations and by protocols approved by institutional animal care and use committee.

### PCR genotyping

Genomic DNA was extracted from tails, whole embryos or yolk sac, and used for genotyping by PCR analysis. For the genotyping of *Cul4b* flox mice, primers p01 (5′-ACAGGTATTTGCCAGTGCTGTC-3′) and p02 (5′-TTCTGTTACCTTCCTACCGAGAG-3′), flanking the loxP site in intron 2, were used to amplify *Cul4b* flox allele (501 bp) and wild-type allele (383 bp) (Fig. S3A). For the genotyping of *Cul4b* null mice, primers p03 (5′-GACTTTACAGAGTTTATCGTTGGT-3′) and p04 (5′-ACAAGAGGGAGATGGTCAGC-3′) were used for detection of the *Cul4b* null allele (498 bp), and primers p05 (5′-AGCACGCAGGCACATAAACG-3′) and p06 (5′-CTGGAACCCCAAGGCAGAAG-3′) were used for detection of the *Cul4b* wild-type allele (321 bp) (Fig. S3B).

### Reverse transcription PCR and real-time RT-PCR

Total RNA from the brain tissues of 2-week mice of different genotypes was isolated using Trizol reagent (Invitrogen, Carlsbad, CA, USA), and treated with RQ1 RNase-Free DNase (Promega) to eliminate genomic DNA contamination. Freshly isolated RNA was reverse transcribed to generate cDNA using Super Script first-strand synthesis system (Invitrogen) following the manufacturer's recommendations. *Cul4b* gene was amplified by PCR using cDNA as template, and PCR product were sequenced. Real-time PCR was performed for quantitation of *Cul4b* mRNA using the TagMan 7500 instrument (PE Applied Biosystems). The mRNA levels of *Cul4b* was measured by SYBR Green I assay using SYBR Green Universal PCR Master Mix (Applied Bilsystems). Mice *Gapdh* was used as endogenous control. The sequences of the primers were for *Cul4b*, 5′-TATTAGTTGGCAAGAGTGCAT-3′ and 5′-CCAGTAACCCATTGTCAGGAT-3′, and for *Gapdh*, 5′-AGGTCGGTGTGAACGGATTTG-3′ and 5′-TGTAGACCATGTAGTTGAGGTCA-3′. Four independent measurements per sample were performed. The quantified individual RNA expression levels were normalized to *Gapdh*.

### Western blot

Protein was extracted from brain tissues of 2-week mice of different genotypes. The concentration of tissue lysates was determined by using the BCA kit (Pierce, Rockford, IL, USA). Then equal amounts (50 µg) of total protein was subjected to 12% SDS-polyacrylamide gel for electrophoresis, followed by blotting onto polyvinylidene difluoride (PVDF) membranes (Amersham Pharmacia Biotech), and incubated with the anti-Cul4b primary antibody (Sigma; used at 1∶1,000 dilution) overnight at 4°C. After washing, the membranes were incubated with a horseradish peroxidase (HRP) conjugated secondary antibody (Jackson ImmunoResearch; 1∶10,000 dilution) for 1 hour at room temperature. Chemiluminescence detection was performed by ECL PLUS kit (Amersham Pharmacia Biotech). The membranes then were exposed to X-Omat Kodak film (Perkin Elmer) to visualize the bands. GAPDH was used as a loading control (Sigma, 1∶5,000 dilution).

### Timed pregnancies

To generate timed pregnancies, female mice were injected intraperitoneally with pregnant mare's serum gonadotropin (PMSG, 5 IU per animal), followed by injection with human chorionic gonadotropin (HCG, 5 IU per animal) 48 hours later and mating overnight with males. The next morning, males were removed and females were examined for the presence of vaginal plugs. Females were sacrificed at 7.5, 8.5, 9.5, 10.5, 12.5 or 14.5 dpc and the embryos were dissected under a dissection microscope.

### Histology

Specimens were dissected and fixed in 4% paraformaldehyde at 4°C overnight, followed by two different processes: (1) tissues of adult mice were cryo-sectioned for immunohistochemistry; (2) embryos were dehydrated, embedded in paraffin, and then sectioned for hematoxylin and eosin (H&E) staining, immunohistochemistry and TUNEL assay.

### Immunohistochemistry

After deparaffinization and rehydration, the sections were boiled in citrate sodium buffer for 15 minutes for antigen recovery, and immersed in 3% H_2_O_2_ for 10 minutes to quench endogenous peroxidase. Sections were then blocked with 10% serum at 37°C for 1 hour. The primary antibodies were added to the sections and incubated overnight at 4°C. The primary antibodies used are anti-Cul4b (Sigma, 1∶1,000 dilution), anti-ki67 (Abcam, 1∶200 dilution), anti-cyclin E (Abcam, 1∶200 dilution), anti-PECAM (Abcam, 1∶50 dilution), anti-Cul4a (Abcam, 1∶200 dilution), anti-p27 (Santa Cruz, 1∶200 dilution), and anti-p53 (Santa Cruz, 1∶200 dilution).

After washing, the sections were coated with a horseradish peroxidase (HRP) conjugated second antibody (Jackson ImmunoResearch; 1∶200 dilution) and then incubated at 37°C for 1 hour. The DAB was used to visualize immunoreactions sites. Sections were counterstained with hematoxylin and mounted on glass slides. Negative controls were obtained by substituting the primary antibody with normal serum.

### BrdU incorporation and immunofluorescence

For labeling of cells in S phase, BrdU (Sigma-Aldrich) was injected intraperitoneally into pregnant mice at 7.5 dpc, with 100 mg per Kg body weight. Animals were sacrificed after 2 hours by cervical dislocation and the embryos were recovered in ice cold PBS and were fixed in 4% paraformaldehyde. Incorporation of modified nucleotide was detected by staining with an anti-BrdU primary antibody (Abcam, 1∶100 dilution) and Rhodamin-labeled secondary antibody (Jackson ImmunoResearch; 1∶100 dilution). After staining, the slides were counterstained with DAPI and visualized under a fluorescence microscopy.

### TUNEL assay

TUNEL assay was performed using the *In Situ* Cell Death Detection Kit, TMR red (Roche) following the manufacturer's recommendations. After labeling, the slides were counterstained with DAPI and visualized under a fluorescence microscopy.

### Statistical Analysis

Data were expressed as the mean±SD. Data from the two groups were evaluated statistically by a two-tailed unpaired t test using SPSS13.0 for any significant differences. A p value of less than 0.05 was considered statistically significant.

## Supporting Information

Figure S1 **Expression of Cul4a, p27 and p53 in wild-type and** ***Cul4b*** **null embryos at 7.5 dpc.** Paraffin sections of wild-type and *Cul4b* null embryos at 7.5 dpc were stained with an antibody against Cul4a (A), p27 (B) and p53 (C). Sections were counterstained with haematoxylin. Lower panels (100×) are the higher magnification of the upper panels (20×).
(TIFF)

Figure S2 **Western blot analysis of Cul4b levels.** Proteins prepared from tissues of wild-type and heterozygous mice at 4 months were subjected to Western blot analysis using an anti-Cul4b antibody. Gapdh was used as a loading control.
(TIFF)

Figure S3 **PCR genotyping of** ***Cul4b*** **flox mice and** ***Cul4b*** **null mice.** (A) PCR genotyping analysis of tail DNA from wild-type (WT), *Cul4b* flox and heterozygous (Het) mice. (B) PCR genotyping analysis of wild-type mice (*Cul4b* ^+/+^ and *Cul4b* ^+/Y^), *Cul4b* knockout male mice (*Cul4b* ^null/Y^) and *Cul4b* heterozygous female mice (*Cul4b* ^+/null^). The null allele can only be amplified by primers p03 and p04 when exons 3–5 are deleted.
(TIFF)
