# Supplementary figures and images for: Lack of Cul4b, an E3 Ubiquitin Ligase Component, Leads to Embryonic Lethality and Abnormal Placental Development
Source: PLoS One. 2012 May 14;7(5):e37070. doi: 10.1371/journal.pone.0037070 (PMC3351389; doi:10.1371/journal.pone.0037070)

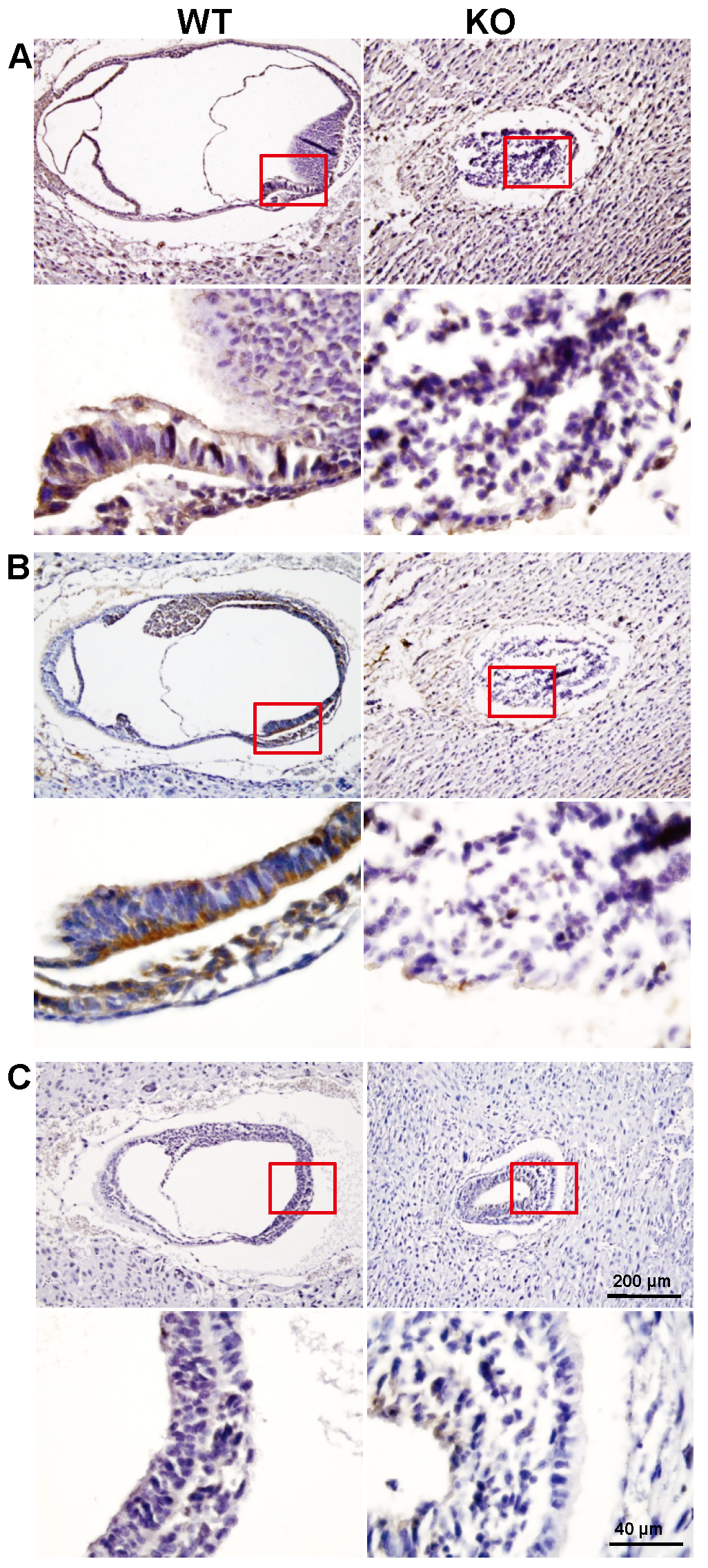

Supplement: Figure S1 — Expression of Cul4a, p27 and p53 in wild-type and Cul4b null embryos at 7.5 dpc. Paraffin sections of wild-type and Cul4b null embryos at 7.5 dpc were stained with an antibody against Cul4a (A), p27 (B) and p53 (C). Sections were counterstained with haematoxylin. Lower panels (100×) are the higher magnification of the upper panels (20×). (TIFF) [file pone.0037070.s001.tiff]

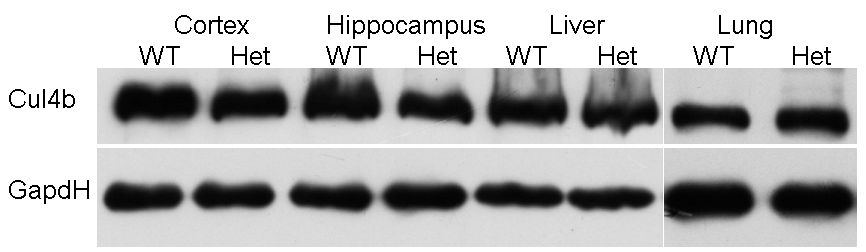

Supplement: Figure S2 — Western blot analysis of Cul4b levels. Proteins prepared from tissues of wild-type and heterozygous mice at 4 months were subjected to Western blot analysis using an anti-Cul4b antibody. Gapdh was used as a loading control. (TIFF) [file pone.0037070.s002.tiff]

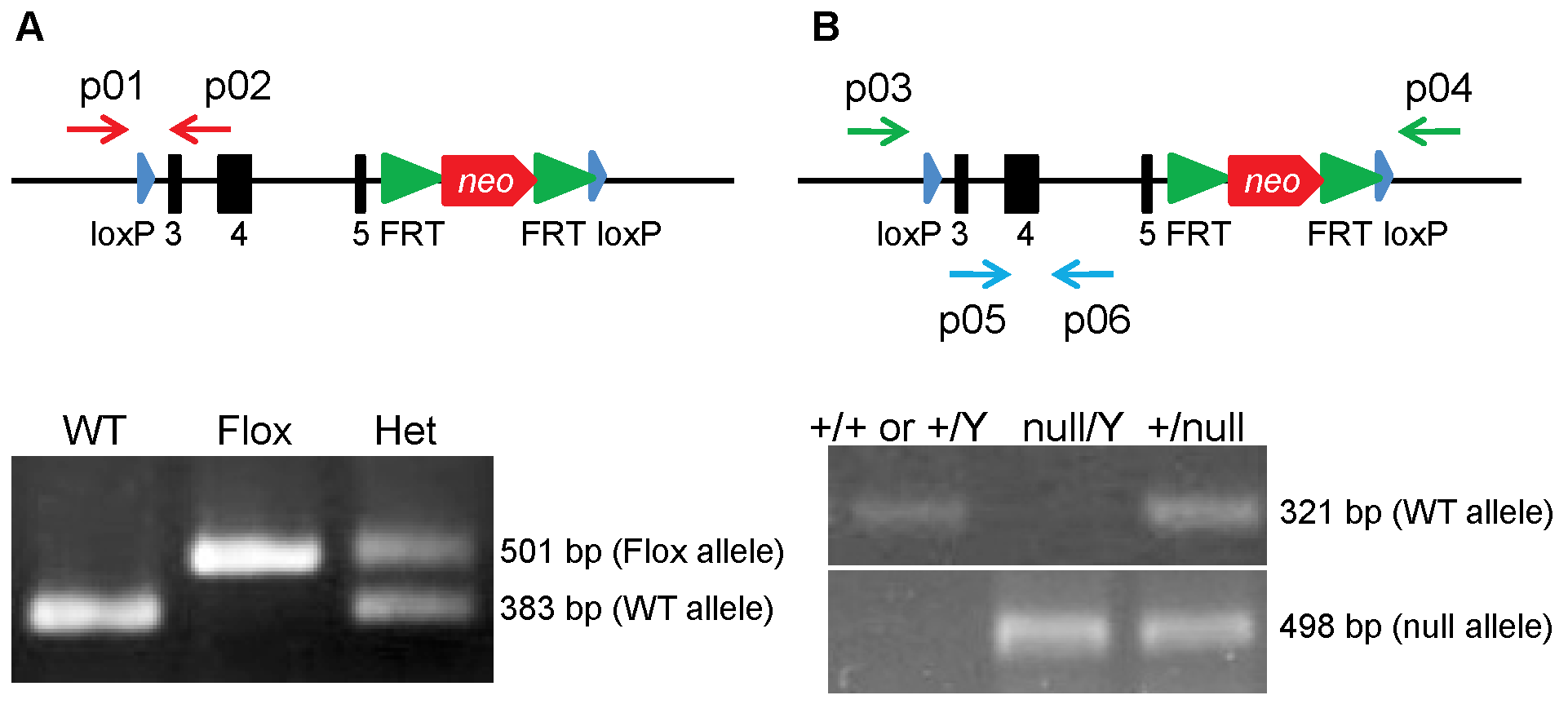

Supplement: Figure S3 — PCR genotyping of Cul4b flox mice and Cul4b null mice. (A) PCR genotyping analysis of tail DNA from wild-type (WT), Cul4b flox and heterozygous (Het) mice. (B) PCR genotyping analysis of wild-type mice (Cul4b +/+ and Cul4b +/Y), Cul4b knockout male mice (Cul4b null/Y) and Cul4b heterozygous female mice (Cul4b +/null). The null allele can only be amplified by primers p03 and p04 when exons 3–5 are deleted. (TIFF) [file pone.0037070.s003.tiff]
